# A bibliometric analysis of published literature on healthcare facilities' wayfinding research from 1974 to 2020

**DOI:** 10.1016/j.heliyon.2022.e10723

**Published:** 2022-09-21

**Authors:** Lujie Deng, Nurul Hanim Romainoor

**Affiliations:** School of the Arts, Universiti Sains Malaysia, Penang 11800, Malaysia

**Keywords:** Healthcare facilities, Wayfinding, VOSviewer, Knowledge structures, Bibliometric analysis

## Abstract

**Objectives:**

Wayfinding in complex environments is a public issue facing the world and has become a hot research topic in recent years. This article reviews and quantitatively analyzes the literature on wayfinding in healthcare facilities and collates the research trends and hotspots in this area.

**Methods:**

The article used bibliometric analysis to search keywords in the Scopus database in the TITLE-ABS-KEY format. In total, 2359 articles were finally collated between 1974 and 2020 after three screening exercises, and a co-citation analysis was conducted by VOSviewer literature visualization analysis software.

**Results:**

Research in the worldwide literature on wayfinding in healthcare facilities has grown steadily year on year since 2002. Computer science (21.5%) and social science (15.5%) are the most common subject categories, with the United States (N = 767) accounting for the largest proportion of research. “Lecture Notes In Computer Science Including Subseries Lecture Notes In Artificial Intelligence And Lecture Notes In Bioinformatics” is the most active journal in terms of publications (N = 169).

**Conclusion:**

Wayfinding cognition, wayfinding behavior, and individual and group differences are currently the focus of research in the field of healthcare facilities. Future research on wayfinding in healthcare facilities will further investigate intelligent assistive technologies and universal designs such as universal signage systems. In addition, cross-cultural-based wayfinding research is a hot topic for future studies where the boundaries of research are broadened and have practical value.

## 1 Introduction

The term "wayfinding" was first coined by Kevin Lynch (Kevin Lynch, 1960) and refers to the human ability to reach a destination in unfamiliar as well as familiar environments (C.-H. Chen et al., 2009). According to Downs and Stea, wayfinding is the ability to orientation, monitor, route selection, and target identification while moving across an environment (Downs et al., 2017). Passini defined wayfinding as the result of spatial cognitive functions (e.g., problem solving and decision making) and behaviors (e.g., navigation and decision execution) (Passini, 1981; Passini et al., 1990). Spatial cognition plays an important role in wayfinding behavior, human can gain the spatial knowledge of new environmental space by crossing the environment (Jansen-Osmann and Fuchs, 2006). Accordingly, the combination of spatial cognitive strategies and the acquisition of spatial knowledge is required for wayfinding, and this type of searching is referred to as wayfinding spatial orientation or spatial navigation (Iftikhar et al., 2020). Orientation allows access to spatial knowledge and cognitive mapping to provide instructions more comprehensively and at a more abstract level. Users learn to investigate knowledge that will enable them to access information about their surroundings and plan routes to advance their spatial orientation and wayfinding (Schwering et al., 2017). The hypothesis of the cognitive map was first proposed by Edward Tolman (1948). Golledge et al. define cognitive mapping as the process of encoding, storing, and changing geo-referenced information that one has experienced or sensed (G. Golledge et al., 2000). According to the cognitive map, the brain creates a unified representation of the spatial environment to aid memory and plan the route during navigation (Epstein et al., 2017). Additionally, the physical environment variables, including the degree of differentiation, the degree of visual access, and the complexity of the spatial layout, will affect spatial orientation and wayfinding (Gärling et al., 1986).

Wayfinding in many complex environments can easily lead to emotional problems such as frustration and anxiety, and emotional problems in turn have an impact on wayfinding behavior and wayfinding performance (Gärling et al., 1986; Lawton and Kallai, 2002a; Passini, 1996). This is particularly challenging in complex environments, such as hospitals, airports, shopping malls, and libraries.

Research on wayfinding in complex environments has evolved over the past 40 years. As researchers have delved into this area of study, a succession of scholars has summarized the research progress in this area. Jamshidi et al. explored the complex factors of wayfinding in indoor environments through a review and emphasized that wayfinding involves human cognitive processes and behaviors. The review study identified two factors that influence wayfinding: (a) user factors and (b) environmental factors (Jamshidi et al., 2020). To explore the extent to which knowledge of the wayfinding domain is established and related concepts, the following section provides a brief review of research related to the field of wayfinding based on these two factors.

User factors include wayfinding cognition, wayfinding behavior, and individual and group differences. The cognitive function, which refers to the mental processes involved in knowledge acquisition, information comprehension, and reasoning, is the focus of a substantial corpus of research on wayfinding (Devlin, 2014; Jamshidi et al., 2020). The gender differences, age differences, and cultural differences of individuals will influence their spatial memory, information acquisition, spatial ability, spatial problem solving, and thus final wayfinding cognition and wayfinding behaviors (Kirasic et al., 1992; Mondschein and Moga, 2018; Passini et al., 1990; Sandstrom et al., 1998; Suzuki, 2013). Studies have shown that age can cause differences in wayfinding cognitive processes, as younger people have better wayfinding performance than older people, and age-specific mental states will also have an impact on wayfinding performance (Barnes et al., 2016; Bosch and Gharaveis, 2017; Kirasic et al., 1992; Ramanoël et al., 2020). In addition, gender differences can affect wayfinding strategies and wayfinding behavior, whereby males and females differed in their acquisition of wayfinding information, spatial memory, and wayfinding problem solving (C.-H. Chen et al., 2009; Lawton and Kallai, 2002a; Martens and Antonenko, 2012). Morag et al. (2016) emphasized that educational background and knowledge structure will affect people's wayfinding behaviors. Andrivina (2019) used a descriptive analysis approach to collect samples to provide solutions to wayfinding design problems related to hospital user characteristics. Rousek and Hallbeck (2011b) contributed by analyzing the existing challenges in wayfinding tasks for visually impaired and normal-sighted people to identify design weaknesses in wayfinding. The above studies have all shown that differences in users' age, gender, and educational background will affect their wayfinding performance in hospital environments. Their research focused on enhancing healthcare facilities' wayfinding through user-centered design. In addition, cultural differences, as well as language and accessibility aspects (e.g., visual impairment, hearing impairment, and physical disability), have also received more attention as they affect a user's processing of wayfinding information.

Environmental factors include environmental elements and environmental cues (Jamshidi et al., 2020). Analyzing how people collect and process environmental information has been a focus in the existing literature to fully understand how users find their way around healthcare facilities (Iftikhar et al., 2020). Studies have emphasized the role of environmental elements, such as space plan, paths, routing, and landmarks (Carpman et al., 1985; O'Neill, 1991; Zhang, 2019), and environmental cues, such as signage, maps, color, shape, typography, and legibility of signs and symbols, on user wayfinding in healthcare facilities (Arthur and Passini, 1992; Gibson, 2009; Passini, 2002; Rodrigues et al., 2019; Tzeng and Huang, 2009). A hospital signage system is regarded as critical environmental information for wayfinding success. Carpman et al. (1985), who combined environmental research with healthcare design, led to the first use of wayfinding in healthcare facilities. The relevance of nomenclature (i.e., how destinations are named), density (i.e., the number of signs), context, position, and visibility have all been recognized as themes that have garnered further focus in the research and helped people learn and put user-centered hospital wayfinding design into practice. Stevenson (1990), Arthur and Passini (1992), Miller and Lewis (1998), Tzeng and Huang (2009), Calori and Vanden-Eynden (2015), and Carpman and Grant (2016) emphasized the importance of a hospital's signage system, especially in the hospital outpatient area, as it is the area with the highest concentration of hospital traffic. In addition, some studies focused on universal hospital signage system design. For example, S Lee et al. (2014) and Joy Lo et al. (2016) showed the use of universal design concepts and principles for hospital signage systems can help more people through improved wayfinding.

The literature review shows that since the 1970s, the number of publications on wayfinding in indoor complex environments has been growing, the research topics have become more specific, and the boundaries of the research scope have been widened. wayfinding in indoor complex environments such as hospitals, supermarkets, airports, libraries, and other large public spaces have received much attention. Firstly, a large number of studies have examined wayfinding subjects from a cognitive psychological perspective, demonstrating that wayfinding difficulties can lead to emotional problems such as anxiety, frustration, and stress, and the resulting negative effects on Wayfinder and wayfinding space management. Wayfinding behavior and wayfinding performance also vary due to individual and cultural differences. Therefore, population-based wayfinding research is a focus of researchers. Second, wayfinding environmental factors involve multidisciplinary research, such as environmental design, environmental psychology, and environmental management. As a large and complex public space, healthcare facilities' wayfinding research has certain universality, but also its own uniqueness, which makes healthcare facilities’ wayfinding a key area of wayfinding research in future research. In addition, with the development of wayfinding aids such as smartphones and wayfinding technologies such as eye tracking, virtual reality, and big data, it has become more prevalent when studying the complexity of wayfinding in real and virtual environments.

Although there is a growing number of publications in this rapidly growing research area, limited efforts have been made to map the evolution and current trends in wayfinding in healthcare facilities. Currently, research addressing indoor complex environment wayfinding is primarily analyzed through systematic literature reviews. These literature reviews provide an exhaustive analysis of a specific topic. As mentioned above, Jamshidi et al., reviewed the published literature on indoor wayfinding and identified two major factors in indoor wayfinding, namely (a) user factors and (b) environmental factors (Jamshidi et al., 2020). Dalton et al. explored that the role of social factors in wayfinding is important for both basic research and applications. As part of wayfinding, other people can play three major roles: co-decision maker, route guide, or environmental cue (Dalton et al., 2019). Hassan Iftikhar presented various factors affecting wayfinding behavior in complex environments, such as identification indicators, environmental familiarity, signage design, and cultural differences, and discussed the contribution of virtual reality and augmented reality technologies in wayfinding (Iftikhar et al., 2020). Parker et al. reviewed the recent literature on wayfinding tools for people with visual impairments in the real world and analyzed their perceptions of the efficacy of wayfinding technologies and their perceptions. Among the existing published literature, there are few systematic reviews of healthcare facilities' wayfinding studies. Through searching online databases, we found two systematic reviews of healthcare facilities' wayfinding studies (Parker et al., 2021). Ann Sloan Devlin provides a brief overview of the relationship between environmental psychology and healthcare facility design through a systematic review (Devlin, 2014). Ita Rodrigues evaluated more appropriate signage system designs for healthcare facilities through a systematic literature review (Rodrigues et al., 2019).

The above studies provide a better understanding of the evolution of wayfinding theory and synthesize the field of indoor wayfinding, which has implications for wayfinding research in the healthcare facility field. However, as previously mentioned wayfinding research is a dynamic and changing multidisciplinary concept, which makes a comprehensive review of the topic and its evolution a challenge. In addition, information about the wayfinding literature is now equally challenging to manage because of its wide scope and distribution across journals, institutions, disciplines, and topics. As a result, healthcare facility wayfinding, as a unique branch of research in the field of wayfinding, requires a more comprehensive literature analysis. Bibliometric analysis is a more scientific and systematic method, which helps to review the published literature on wayfinding research and the concept evolution over time. The number of publications using bibliometric analysis as a tool for scientific research has been growing in recent years. It has the advantages of handling a large volume of literature, intuitive visualization, a variety of analytical perspectives, and high credibility of data analysis results, compensating for the shortcomings of traditional literature review studies (Kessler, 1963; Yu et al., 2021). Bibliometric analysis is seen as an effective method for assessing scientific results, with advantages in predicting forward trends in the discipline (Wang and Su, 2020). It is becoming increasingly influential (Ellegaard and Wallin, 2015).

A search of the available literature has revealed that bibliometric analysis is less applied in the field of complex environment wayfinding research, especially in specific large indoor environments such as shopping malls, airports, hospitals, and libraries. Ghamari and Sharifi (2021) provided a broad review of the general trend of peer-reviewed publications in the field of indoor wayfinding, pointing out that among specific indoor environments, in addition to spatial knowledge acquisition and cognitive maps, wayfinding visual cues such as signage, as well as the use of wayfinding assistive technologies such as eye-tracking and virtual reality, still require further research. Krey et al. (2022) provided a comprehensive assessment of Jean-Charles Chebat's contribution to the shopping center literature from a management perspective and suggested that future research should enhance shopping center wayfinding by exploring new technologies and assessing potential gender differences to improve the consumer shopping experience.

A study of previous works is a meaningful way to track the development of the discipline and is essential for assessing the development process of the field, grasping the direction of development, and gaining insight into future research hotspots (Yu et al., 2021). Therefore, this study used the bibliometric analysis method to conduct a comprehensive quantitative analysis and a broad review of publications in the field of healthcare facilities' wayfinding. This approach helps us to understand the research trends and key hotspots in the field of wayfinding in complex healthcare facilities and their interactions among keywords, countries, authors, and disciplinary categories. The findings of this study add to previous research that has attempted to depict the field's thematic evolution and support researchers in better understanding the current status of wayfinding in healthcare facilities as well as future research directions.

This study aimed to analyze the structure of knowledge in the field of wayfinding in healthcare facilities by (1) reviewing a large number of publications (2,359 documents); (2) analyzing research trends and hotspots in the field since 1974, identifying keywords, authors, countries, scientific discipline categories, and mapping social network visualization; and (3) exploring future research directions and priority recommendations.

## 2 Method

### 2.1 Database selection

Quantitative and qualitative analyses were conducted through econometric analyses retrieved from large individual databases, such as Scopus and Web of Science (Engwall et al., 2014). In this study, Scopus, the world's largest database of peer-reviewed literature abstracts and citations from the Elsevier Group, was used as the search database. Scopus has the advantage of visually analyzing the literature and providing metrics straightforwardly and simply (Falagas et al., 2008). Therefore, Scopus was chosen for two main reasons: (1) it covers a large area of high-quality research literature and is widely recognized in academia for archiving high-quality peer-reviewed research; and (2) the bibliographic output generated by the database facilitates text mining and bibliometric analyses.

The study period was limited from the years 1974–2020. The literature in the field of wayfinding research in healthcare facilities was reviewed by performing text mining, scientometric analysis, and social network visualization (Saheb and Saheb, 2019). Afterwards, this study discovered "topics, hotspots, and evolution of wayfinding research in healthcare facilities over time, and then analyzed future research topics and trends". This study analyzed and mapped three networks: co-occurrence of keywords, author co-citation network, and country co-citation network. Text mining is developing as a potent approach for harnessing the power of unstructured textual data in big data analytics by analyzing it to extract new knowledge and uncover significant patterns and correlations buried within the data (Hassani et al., 2020; Saheb and Saheb, 2019). In the healthcare facilities' wayfinding field, keyword co-occurrence networks as part of the citation context-based bibliometric networks may also discover differences and similarities in knowledge structures and substructures (Bornmann et al., 2018).

### 2.2 Analysis tools

This study used VOSviewer (version 1.6.16), a visual literature analysis software developed by the Centre for Science and Technology at Leiden University in the Netherlands, as a co-citation analysis tool for the construction and analysis of bibliometric maps (van Eck and Waltman, 2009) and SPSS (version 22.0) for descriptive statistical analysis. The exported data and some of the statistical analysis graphs were created using Microsoft 365. Global distribution maps of publications were created using Mapchart (https://mapchart.net/world.html).

### 2.3 Search strategy

The literature search was conducted on April 25, 2021, using the Scopus database. A search of the digital archives revealed that the first literature on wayfinding research in the available data repositories was published in 1974. In order to more systematically study the current status and evolutionary trends of research in the field of wayfinding, this study collected and organized the literature between 1974 and 2020. The existing literature reviews and bibliometric analyses typically limit the search to the type of article; given that this limitation may not retrieve all relevant publications, it may result in the omission of some important literature. To retrieve as many important publications as possible in the field of wayfinding research in healthcare facilities, the results of the literature search were screened for the following three types of analysis: (1) Article; (2) Conference Paper; and (3) Review. Grey literature, books, book chapters, and notes were excluded.

Wayfinding is a broad field of study that can be divided into indoor wayfinding and outdoor wayfinding in terms of the subject matter. Applied research on wayfinding in healthcare environments typically comes from other institutional settings such as large shopping malls (Krey et al., 2022), airports (Bosch and Gharaveis, 2017a), and libraries (Li and Klippel, 2012). From a planning configuration and signage perspective, wayfinding studies cover any large building or building complex applications to the healthcare environment (Devlin, 2014). Therefore, this study has developed two search themes, healthcare facilities, and wayfinding. Once the research topic has been identified, it is necessary to understand the key terms in the field and create a logic network or concept map (Aromataris and Riitano, 2014). The keywords form a search strategy for both topics. A broad literature search string containing terms relating to healthcare facilities and wayfinding was employed for this purpose. Some keywords were taken from systematic literature reviews, and some were extracted from web searches.

"TITLE-ABS-KEY" (i.e., "title-abstract-keyword") was used as the search formula. Thus, if a keyword is present in any document's title, abstract, or keywords, then the relevant publication is displayed in the search results. The following is the search string used in this study: TS = ((“healthcare facilities” OR “medical facilities” OR “hospital” OR “hospital facilities” OR “medical institutions” OR “healthcare institutions” OR “hospital institutions” OR “medical space” OR “healthcare space” OR “hospital space” OR “medical settings” OR “healthcare settings” OR “hospital settings” OR “medical environment” OR “healthcare environment” OR “hospital environment” OR “Indoor” OR “interior” OR “wayfinding” OR “way∗finding” OR “guide” OR“wayfinding design” OR “way∗finding design” OR “wayfinding system” OR “way∗finding system” OR “guide system” OR “signage” OR “signage design” OR “signage system” OR “navigation” OR “orientation”)).

### 2.4 Data analysis and visualization

The search results were exported as a CSV file named "scopus.csv." The analysis unit type was a co-occurrence-all keyword, the counting method was full counting, and the VOSviewer counted 10,042 keywords according to the exported literature. The node filtering criteria were the frequency of occurrence of nodes ≥3, and the top 1,717 keywords in the node degree ranking reached the threshold, which was about 20% of the total nodes.

This study employed the software SPSS for descriptive statistical analysis and the exported data. Some of the statistical analysis charts were created using Microsoft 365. The search data were exported from Scopus to Excel for statistical and analytical purposes. The exported data included author name, title, year, country and region, journal name, document types, subject categories, the number of publications per year, the number of citations per year, affiliation, author keywords, index keywords, and the language of documents. The mean and standard deviation of the number of citations per publication (CPP) were calculated.

The files were imported into VOSviewer, and the layout and clustering parameters were adjusted to obtain better clusters and layouts. The final data were exported for analysis in conjunction with the plots. VOSviewer provides visual views including three types, Network Visualization, Overlay Visualization, and Density Visualization (van Eck and Waltman, 2009).

## 3 Results

### 3.1 Document type, subject categories, and language of publication

In total 2359 literature items were retrieved according to the search strategy. The majority of document types were research articles (1426, 60.4%) followed by conference papers (834, 35.4%), and then reviews (99, 4.2%). Table 1 shows the subject categories of the documents and the frequency number. The subject category with the highest proportion of documents was "Computer Science" (21.3%), followed by "Social Sciences" (14.7%), and "Engineering" (13.7%). Subject categories with more than 100 documents include mathematics, earth sciences, neurology, arts and humanities, and geography. At the same time, other categories are less represented in the Scopus database. The publication language is mainly English (2317, 98.2%), followed by Chinese (12, 0.005%), and French (10, 0.004%).

**Table 1:** Subject categories focusing on healthcare facilities’ wayfinding (1974–2020).

Subject categories	Frequencies(n)	Percentage (%)
Computer Science	916	21.3
Social Sciences	632	14.7
Engineering	589	13.7
Medicine	395	9.2
Psychology	308	7.2
Mathematics	272	6.3
Earth and Planetary Sciences	202	4.7
Neuroscience	162	3.8
Arts and Humanities	154	3.6
Environmental Science	130	3.0
Other	500	12.7

### 3.2 Trends of publications and citations

**Figure 1:**
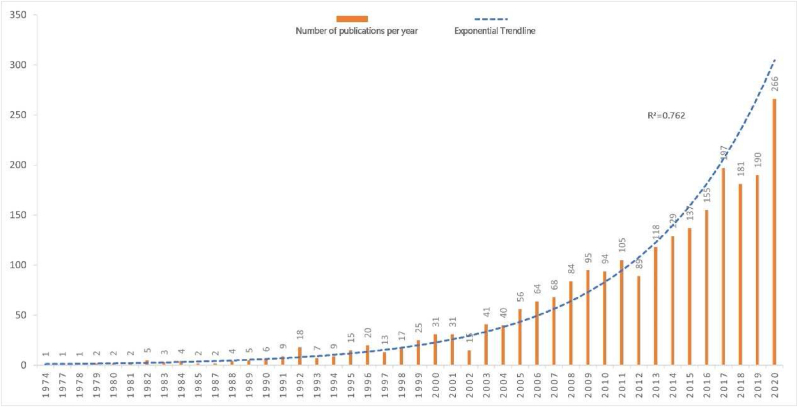
Trends of publications on healthcare facilities' wayfinding (1974–2020).

The trends of publications on healthcare facilities’ wayfinding from 1974 to 2020 are shown in Figure 1. The first article on this topic was published in 1974, with scarce papers produced before 1992 and a dramatic increase in the number of publications published after 2002. The highest number of articles was published in 2020 (266 papers, 11.3%), with more than 35% of the literature review published in the last five years (2016–2020). During the research period, the number of publications R^2^ coefficient increased exponentially, with the number of publications R^2^ = 0.762 and a lower than the predicted number of published literature in 1993 and 1994.

This study analyzed ten articles with the highest number of citations, with the most cited article being “Anonymous usage of location-based services through spatial and temporal cloaking” (N = 1552). These ten articles were all cited more than 300 times, with an average citation count of 564.1. These articles were published between 1994 and 2012, and most were published between 2000 and 2012 (N = 7). The highest number of publications countries were the United States (N = 7), the United Kingdom (N = 2), and Germany (N = 1).

**Figure 2:**
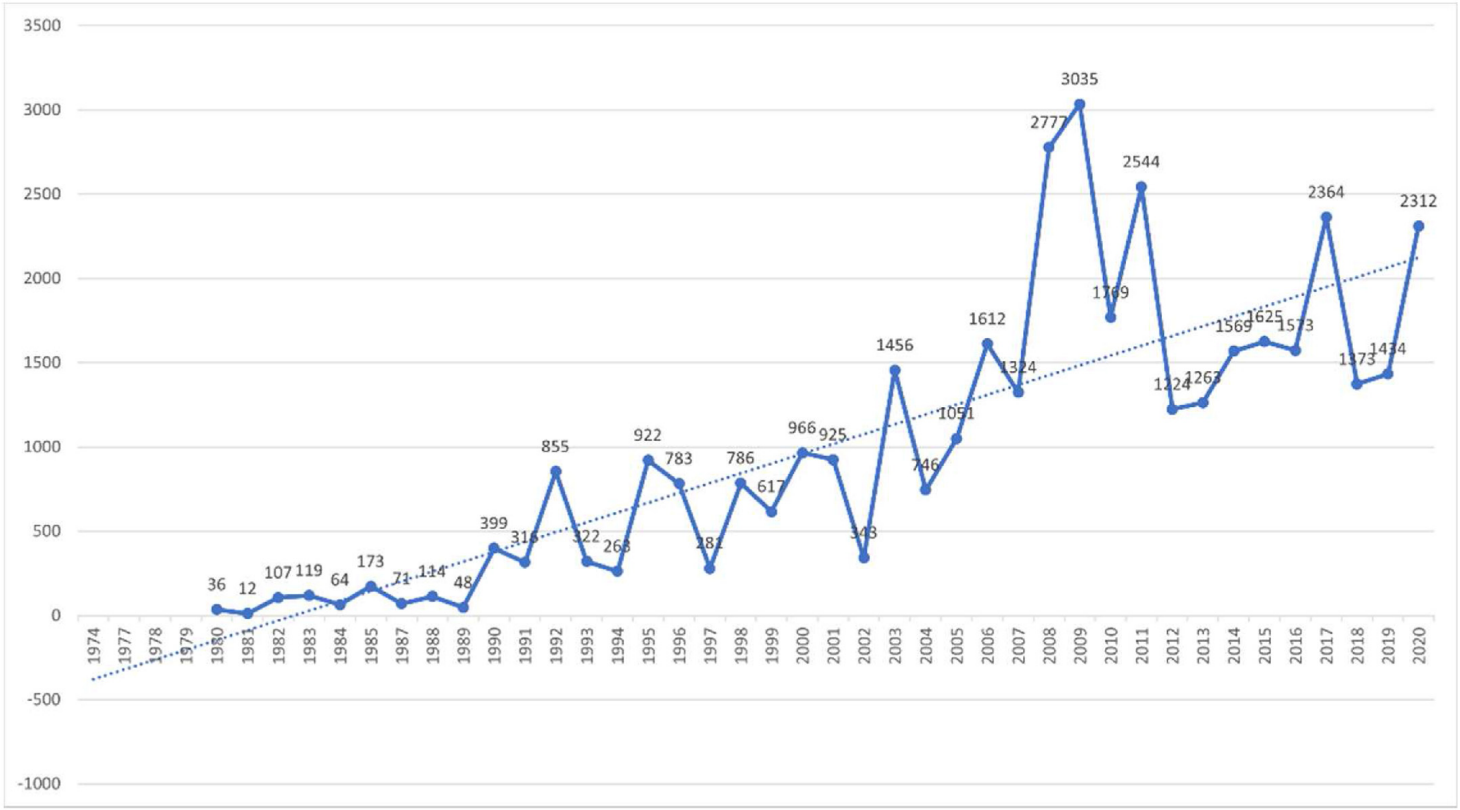
Trends of citations on healthcare facilities' wayfinding (1974–2020).

The trends of citations on healthcare facilities' wayfinding from 1974 to 2020 are shown in Figure 2, literature citations began to appear in 1980, and the number of citations per year has been increasing since 1982. The overall trend can be divided into two phases. The first phase is from 1980 to 2007, and the second phase is from 2008 to 2020, with a peak in 2007 (N = 2777). Since 2003, the number of citations on this topic has been more than 1000 citations per year.

### 3.3 Most active and cited journals

Between 1974 and 2020, there were 1,176 journals with articles related to wayfinding research in healthcare facilities. The Top-10 journals with the largest number of publications are in Table 2, representing approximately 20% of the total number of publications. Lecture Notes in Computer Science Including Subseries Lecture Notes in Artificial Intelligence and Lecture Notes In Bioinformatics (N = 151), Journal of Environmental Psychology (N = 50), and Advances in Intelligent Systems and Computing (N = 37) accounted for the highest number of articles. The journal with the highest impact factor was Environment and Behavior (8.8, 2019).

**Table 2:** Top-10 journals with the largest number of publications.

No.	Name of journal	Number of documents	Percentage	Citations	CPP(citation per publication)
1	Lecture Notes in Computer Science Including Subseries Lecture Notes in Artificial Intelligence and Lecture Notes in Bioinformatics	151	6.40%	2571	17.02
2	Journal of Environmental Psychology	50	2.11%	3119	62.38
3	Advances in Intelligent Systems and Computing	37	1.57%	32	0.86
4	Spatial Cognition and Computation	30	1.27%	1232	41.06
5	Conference on Human Factors in Computing Systems Proceedings	28	1.19%	747	26.68
6	Lecture Notes in Geoinformation and Cartography	28	1.19%	146	5.21
7	ACM International Conference Proceeding Serie	27	1.14%	337	12.48
8	Environment and Behavior	25	1.06%	2128	85.12
9	Frontiers in Psychology	21	0.89%	317	15.10
10	Health Facilities Management	19	0.81%	36	1.89

The Journal Of Environmental Psychology was the most cited journal (N = 3119), followed closely by *Lecture Notes in Computer Science. Including Subseries Lecture Notes in Artificial Intelligence and Lecture Notes in Bioinformatics* (N = 2571), and *Environment and Behavior* (N = 2128) (Table 2).

### 3.4 Keyword co-occurrence analysis

**Figure 3:**
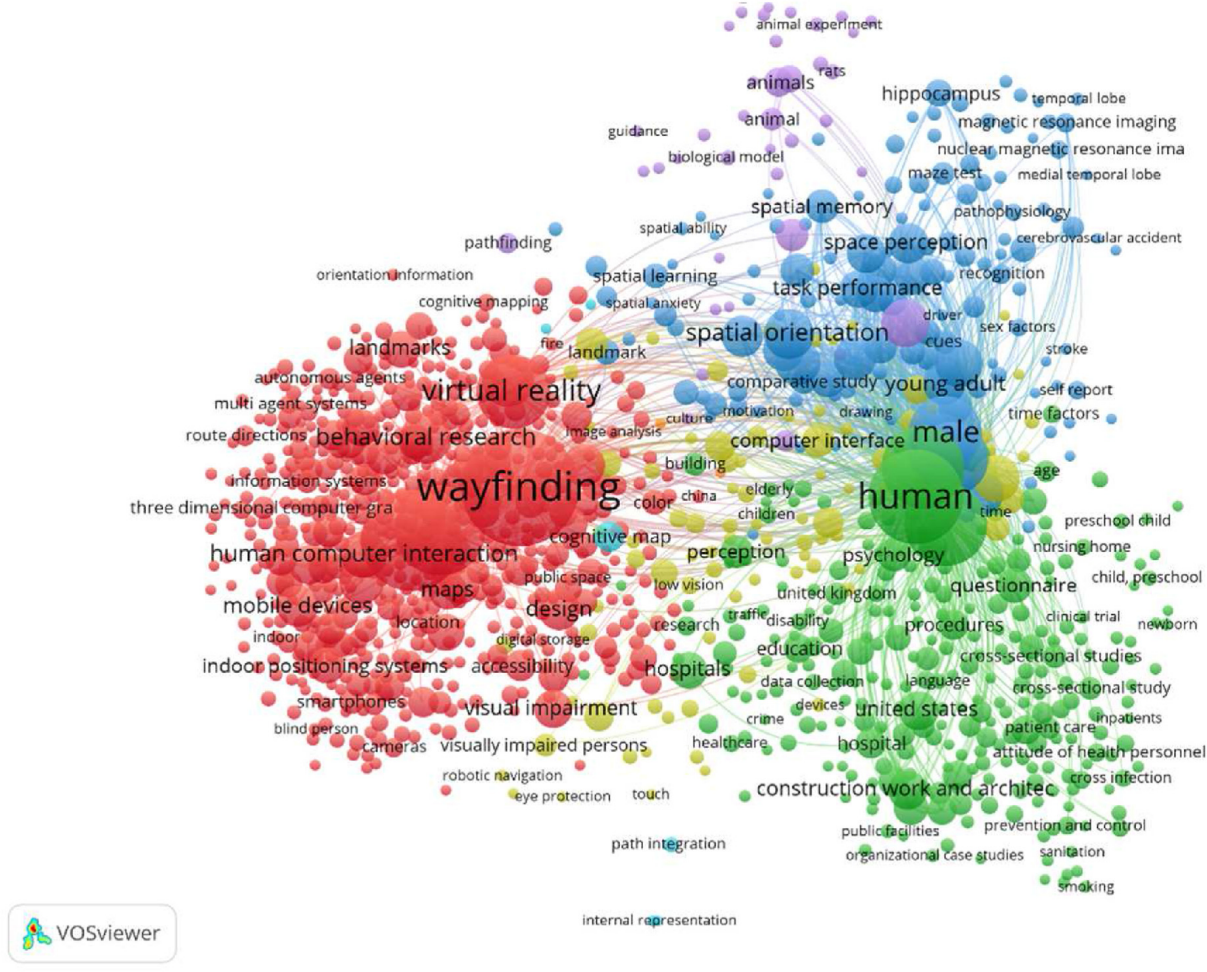
Visualization keywords' co-occurrence network.

The visualization keywords’ co-occurrence network are displayed in Figure 3, the frequency statistics of keyword co-occurrences by VOSviewer, the high-frequency keywords for research in the field of wayfinding between 1974 and 2021 include human, wayfinding, wayfinding systems, navigation systems, gender, virtual reality, maps, spatial positioning, spatial perception, human-computer interaction, orientation, cognition, decision making, design, spatial navigation mobile devices, spatial perception, user interface, signage, and guidance system design. The high-frequency keywords reflect the current hotspots in the research field. Co-occurrence analysis was performed on keywords that appeared more than five times (1096 in total).

#### 3.4.1 Cluster ① (red)

The main research keywords include wayfinding behavior and strategies (Hölscher et al., 2006; Lawton, 1996a; Lawton and Kallai, 2002), spatial cognition (Hölscher et al., 2011; Mondschein and Moga, 2018; Passini et al., 1990), wayfinding system design (Pan and Yin, 2015; Passini, 1996), environment design, guidance system design (Vinson, 1999), indoor navigation (Afyouni et al., 2012), solution of wayfinding problems for blind and visually impaired people (Kulyukin et al., 2006), information design (Passini, 1996), and human-machine interface interaction design (Gruteser and Grunwald, 2003; Liu et al., 2008; Passini et al., 1990), focusing on the classification of wayfinding problems and the differences in influencing factors and solutions to wayfinding problems.

#### 3.4.2 Cluster ② (green)

The main research areas include service and management in healthcare institutions (Flores et al., 2008; Suess and Mody, 2018), hospital orientation design (Seunghae Lee et al., 2014; Rousek and Hallbeck, 2011a, 2011b), and hospital wayfinding (Carpman et al., 1985; Rousek and Hallbeck, 2011b), as well as psychological differentiation among different groups of people (age, gender, and disabled), with many articles using experimental, research, and quantitative analysis methods.

#### 3.4.3 Cluster ③ (blue)

Topics include individual wayfinding behavior, psychological analysis, and spatial orientation (Lawton, 1996), memory confusion (Burgess et al., 2006), research on cross-cultural differences in wayfinding and spatial memory, and cognitive differences in different populations (Lawton and Kallai, 2002a; Montello et al., 1999; Postma et al., 2004) (age and gender) and neuroscientific tests (Occhialini et al., 2016), with research methods mainly based on data analysis through experiments (Xie et al., 2012).

#### 3.4.4 Cluster ④ (yellow)

Research topics include wayfinding behavior and cognitive problems and solutions in special populations (elderly people, people with Alzheimer's disease, people with disabilities, and people with visual impairment) (Passini et al., 2000).

#### 3.4.5 Cluster 5 (light purple)

It focuses on the intersection of wayfinding with neuroscience and biology.

### 3.5 Author co-citation analysis

A total of 5052 authors contributed to the 2359 published documents, with an average of 2.14 authors per document. Multi-author collaborations (≥two authors) accounted for approximately 90% of the documents retrieved, while the rest were single authors. The top ten authors are from nine different countries and institutions around the world. Of the top ten authors in terms of the number of published documents, Hölscher C. was the author with the highest number of published documents (N = 34). Raubal M. was the author with the highest number of citations to the literature, with a total of 1136 citations, and CPP (citation per publication) is 63.11 (Table 3).

**Table 3:** Top ten authors with the highest number of publications and citations.

NO.	Author	Country	Institution	Number of documents	Citations	CPP
1	Hölscher C.	Germany	Forschungszentrum Borstel - Zentrum für Medizin und Biowissenschaften, Borstel, Germany	34	1084	31.88
2	Raubal M.	Switzerland	Institute of Cartography and Geoinformation, ETH Zürich, Zürich, Switzerland	18	1136	63.11
3	Richter K.F.	Sweden	Department of Computing Science, UmeÃ1 University, Sweden	17	259	15.23
4	Winter S.	Australia	Department of Infrastructure Engineering, The University of Melbourne, Australia	16	958	59.88
5	Dalton R.C.	United Kingdom	Faculty of Engineering and Environment, Northumbria University, Newcastle upon Tyne, United Kingdom	15	375	25.00
6	Manduchi R.	United States	Dept. of Computer Engineering, University of California, Santa Cruz, United States	14	298	21.29
7	Schwering A.	Germany	Institute for Geoinformatics, University of Muenster, Heisenbergstr	14	146	10.43
8	Gartner G.	Austria	Department of Geodesy and Geoinformation, Vienna University of Technology, Vienna, Austria	13	184	14.15
9	Passini R.	Canada	Centre de Recherche, Inst. Univ. Geriatrie Montreal, Montreal, Canada	13	858	66.00
10	Rebelo F.	Portugal	Faculdade de Arquitetura, Universidade de Lisboa, Portugal	13	259	19.92

**Figure 4:**
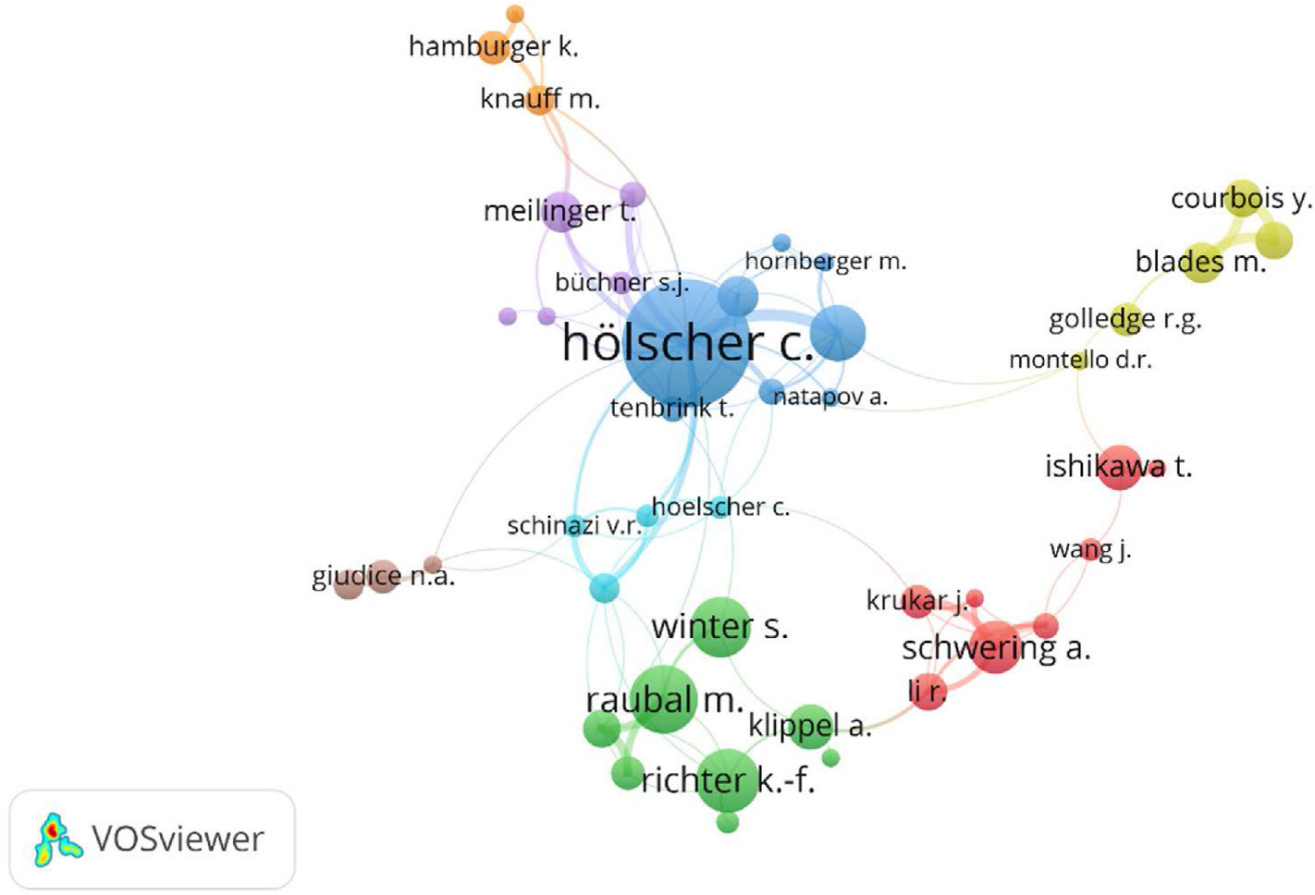
Visualization of author co-citation network.

The most prominent authors in the healthcare facilities' wayfinding literature were identified by a co-citation analysis employing cited authors. To optimize the data results, the minimum number of author publications was set to 3, and the minimum number of citations per author to 10 in VOSviewer; 364 of the 5052 authors reached this threshold, resulting in author co-citation network clustering. As shown in Figure 4, a circle in the figure represents an author node. The circle size indicates the number of publications and the number of citations of the author's literature; here, the higher the number of citations is, the more significant it is. Nodes are linked to each other to indicate co-citation relationships. The most active authors can be identified in 8 clusters, according to their expertise. The findings are mostly consistent with those of the preceding section's analysis (authors with the highest number of publications and citations).

The Blue cluster authors mainly focused on wayfinding behavior and the use of cognitive, spatial syntax, and virtual navigation techniques including Hölscher, C.; Dalton, R.C.; Wiener, J.M.; Kuliga, S.; and Tenbrink, T., et al. The Green cluster authors mainly focused on the development of novel navigation assistance technologies and wayfinding models, such as eye-tracking technologies, mobile cognition, and mobile geosensor networks. Authors included Raubal, M.; Winter, S.; Richter, K.F.; Klippel, A.; and Schmid, F. The Red cluster authors focused on the influence of individual differences and environmental factors on wayfinding performance. They included Schwering, A.; Ishikawa, T.; Krukar, J.; Wang, J. et al. The authors of the light green cluster focused on wayfinding behavior and wayfinding problem-solving in special populations, such as children, Williams syndrome, intellectual disability, and Down syndrome. The authors included are Courbois, Y.; Blades, M.; Golledge, R.G.; and Montello, D.R.

### 3.6 Country co-citation analysis

**Figure 5:**
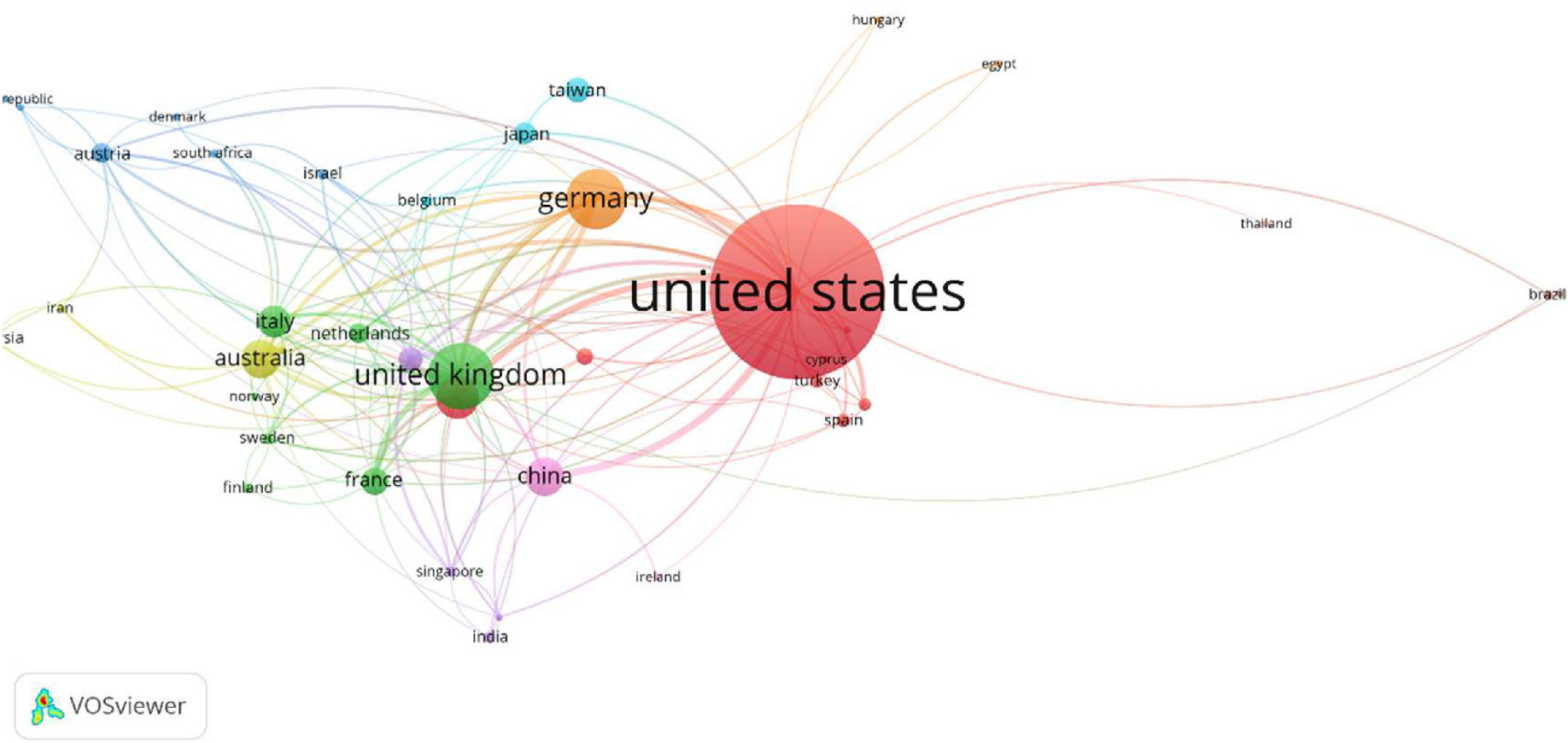
Visualization of country co-citation network.

Country co-citation analysis identifies the most influential countries that contribute the most to knowledge in the healthcare facilities' wayfinding field. The results of the bibliographic analysis of the most active countries are shown in Figure 5. The size of the nodes is proportional to the number of publications, and the thickness of the line connecting the countries represents the strength of the research collaboration.

**Figure 6:**
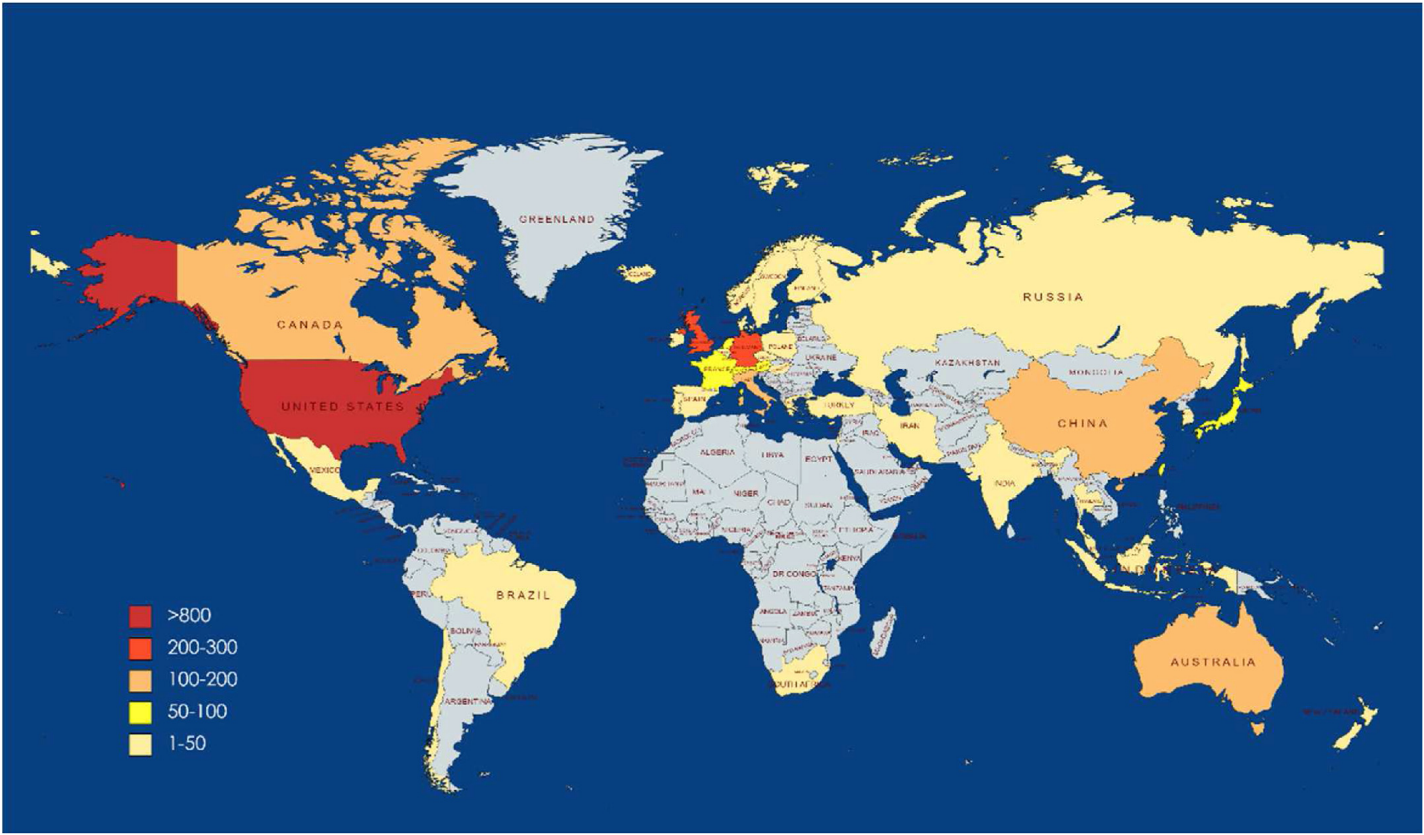
Global map of literature published in different countries.

The literature in the healthcare facilities’ wayfinding field came from 81 different countries. A global map of the countries where the literature was written is shown in Figure 6. Among all these countries, three countries published more than 200 papers, accounting for 51.2% of the total published literature: the United States published the most significant number of papers (N = 817), and similarly, papers from the United States received the most significant number of citations (N = 19068), the United Kingdom (N = 252) and Germany (N = 217) cited (N = 5056) and Germany (N = 4244), respectively. Four countries produced more than 100 publications (Canada, Australia, China, and Italy).

It can be seen that the United States, the United Kingdom and Germany may be particularly interested in further research into salient issues in the field of wayfinding and the ongoing search for solutions and strategies to problems. In addition, the Swiss Federal Institute of Technology (ETH Zürich-Eidgenössische Technische Hochschule Zürich) and the University of Melbourne (Australia) are the two leading research institutions of most published, followed by the Technical University of Vienna (Austria) and the University College London (UK).

## 4 Discussion

The findings of this study may provide insights into the evolution of the literature on healthcare facility wayfinding and provide a better understanding of healthcare facilities' wayfinding research trends, tools, techniques, and methods. According to the analysis, the number of publications on healthcare facilities' wayfinding research has shown a continuous increase from 1974 to 2020. Of all the literature reviewed, the main language of publication is English, and the publication type is predominantly research articles. The country with the highest number of publications is the United States, accounting for 35% of the total number of publications. In addition, the study of healthcare facilities' wayfinding is multidisciplinary, involving not only cognitive psychology, environmental science, neurology, anthropology, linguistics, architecture, animal behavior, and artificial intelligence, but also computer science, management, and other fields.

Since the early stages of the development of wayfinding research in healthcare facilities, keywords such as navigation, architectural and engineering environment design, guidance and signage, spatial cognition, wayfinding behavior, and hospital design, have appeared with high frequency. Research in this phase focused on the human wayfinding process as behavioral and cognitive research and architectural environment design. Since 2007, the field of wayfinding in healthcare facilities has entered a new era of exploring wayfinding assistive technologies. More research focused on wayfinding assistive technology. Keywords such as virtual reality, mobile devices, user interface, navigation systems, and human-computer interaction have gradually emerged. Wayfinding system design in healthcare facilities is also the main focus of research, as keywords for wayfinding systems and signage systems have increased in frequency. With the emergence of intelligent wayfinding assistive technologies and devices, new research on technology adoption and behavior change is being conducted to improve wayfinding and healthcare management in healthcare facilities.

In general, the hot trends in the field of wayfinding research in healthcare facilities are generally characterized by the following.

First, researchers are continuing to focus on the wayfinding behavior and wayfinding performance of specific populations. They have explored solutions to the difficulties of wayfinding caused by differences in spatial perception, by analyzing specific populations such as older adults and children, male and female, special groups such as those who are visually impaired and autistic, and those with dementia and disabilities. Population aging is a significant trend worldwide, and studies have shown that age can cause differences in spatial cognitive processes (Kirasic et al., 1992). Thus, there is a growing interest in wayfinding among the elderly population (Bosch and Gharaveis, 2017a; Marquez et al., 2017; Ramanoël et al., 2020; Webber and Charlton, 2001) as well as the development of navigation devices and tools for the elderly population to address wayfinding difficulties (Barnes et al., 2016; Grierson et al., 2009; Seunghae, 2013; Webber and Charlton, 2001; Wolstenholme, 2010). Wayfinding strategies have gender differences (C.-H. Chen et al., 2009; Lawton and Kallai, 2002). There are similarities and differences in the visual behavior of men and women during the process of wayfinding (Dong et al., 2020), as women rely mainly on landmark information during wayfinding, while men are more likely to use both landmark and geometric information (Sandstrom et al., 1998). Navigation tools that collect data on the behavioral and cognitive underpinnings of the user can reduce gender differences in the process of wayfinding (Martens and Antonenko, 2012). In addition, navigation tools for people with visual impairments (Nam et al., 2015; Oliveira et al., 2018; Papadopoulos et al., 2020; Rey-Galindo et al., 2020), people with autism (Irish, 2019; Yang et al., 2021), and people with dementia (Blackman et al., 2007; Cm et al., 2020; Gresham et al., 2019; Marquardt and Schmieg, 2009) are increasingly being studied for wayfinding solutions and techniques.

Second, emphasis has been placed on the research of wayfinding assistive technologies and guidance systems in healthcare facilities. The design of hospital wayfinding systems, virtual reality, and indoor navigation technologies have gradually received attention. For example, virtual reality technology has been used to simulate hospitals in order to assess the wayfinding abilities of elderly people with early dementia (Jiang and Li, 2007). Cognitive maps for older and younger people have been developed in simulated virtual hospital environments (Kumoğlu and Olguntürk, 2016). Research has also been conducted to create user-centered mobile application designs for hospital wayfinding systems (Harper et al., 2020; Harper et al., 2020). Indoor positioning in hospitals through mobile devices (e.g., smartphones) (Hou et al., 2018) and improved hospital signage design can help solve people's wayfinding problems (Eden Jayne Short et al., 2017; Eden J. Short et al., 2019). Smart touchscreens and digital signage in healthcare facilities also offer practical solutions to wayfinding problems (Pianalto, 2016). Landscape gardening can also influence individuals to find their way around healthcare facilities (Zabihi et al., 2021).

There is increasing interest in cross-cultural studies in the field of healthcare facilities' wayfinding. The aim is to investigate inclusive and accessible wayfinding designs by examining cross-cultural variations in wayfinding. Studies have shown that cultural differences influence wayfinding strategies and the performance of wayfinding (Lawton and Kallai, 2002a), and that cultural differences exist in human spatial perceptions during wayfinding (Suzuki, 2013). People's age and gender influence their wayfinding strategies and behaviors (Morag et al., 2016). One study shows that people over 60 years have a lower willingness to use mobile apps for navigation, and the need for improved navigation (including mobile apps) positively correlates with education level (Ženka et al., 2021). People's educational background and knowledge structure also have an impact on wayfinding behaviors (Morag et al., 2016,), and different cultural backgrounds result in differences in the comprehension of the wayfinding visual aid such as healthcare symbols (Joy Lo et al., 2016; S Lee et al., 2014; Wendy T. Olmstead, 1999). Designers should consider the cultural limitations of signage interpretation during the design process (Hashim et al., 2014) and develop and test generic healthcare symbols that can be used in different countries, such as Inclusive design and Universal healthcare symbol design to enhance the multilingual accessibility of hospital signage (Schuster et al., 2017). Cultural differences and their effect on perceiving environmental components such as signage, maps, and directories, as well as spatial layout indicate that additional research is required in developing nations and diverse environments.

This study investigated the current status and evolutionary trends of peer-reviewed publications in the field of wayfinding in healthcare facilities by using a bibliometric analysis method, and the results of this study complement previous studies. However, this study also has certain limitations as follows. (1) Although the bibliometric analysis method can help researchers provide a higher level of analysis of research trends, productivity in different fields, and patterns of disciplinary connectivity in an increasing number of articles (Ellegaard and Wallin, 2015), it focuses more on synthesizing topics and mapping knowledge, and so it cannot replace the systematic literature review. Future research is necessary to perform in-depth systematic reviews of wayfinding studies on specific topics and fields. (2) Although the Scopus database is one of the most comprehensive and integrated subject databases available, in some countries such as those in Asia and Africa, peer-reviewed journals are not indexed by Scopus, and therefore some publications may be missed for review. (3) Due to the language proficiency limitations of the authors of this study, we only analyzed publications written in English when conducting the literature analysis, papers that were not written by researchers in English may have been overlooked.

## 5 Conclusion

This paper analyzes the exploration and practice in the field of wayfinding in healthcare facilities and provides some ideas and references for future research on wayfinding in healthcare facilities. First, cross-cultural research in the field of wayfinding in healthcare facilities should be strengthened to further investigate the differences in user perception and understanding due to cultural differences and to explore the personal and environmental factors in the process of wayfinding in healthcare facilities. As the results of this study show that the most active countries and institutions are mostly from developed countries, the contribution of Asian, African, Middle Eastern, and Eastern European countries is relatively low. Considering the cultural differences, it is prudent to consider whether the current study is representative or useful for these countries and regions, and so studies from these countries and regions deserve to be explored in depth. In addition, "universal design" and "inclusive design" in wayfinding are trends based on globalization, and cultural consistency and comprehensibility in wayfinding visual aids can enhance a user's understanding of the environment and reduce the difficulty and stress of wayfinding. For example, the design of universal healthcare symbols needs further study. Second, the development of emerging technologies (e.g., digital wayfinding, augmented reality, virtual reality) and intelligent wayfinding tools (e.g., mobile apps) for the study of wayfinding in healthcare settings could be used to explore human perceptions and preferences and improve wayfinding difficulties through the role of wayfinding assistive technologies and tools. Third, future research could be more focused on investigating solutions to wayfinding challenges for people with special needs such as children, disabled people, visually impaired people, and those with Alzheimer's. Especially with the global trend of aging, older adults are the ones to focus on, and they are frequent visitors to hospitals. Studies have shown that the elderly are more likely to get lost and have worse wayfinding performance than younger people due to cognitive and visual decline (Aboim Borges & Da Silva, 2015; Devlin, 2014; Zijlstra et al., 2016). Therefore, how to solve the problem of elderly people's hospital wayfinding is a key direction for current and future research.

## Declarations

### Author contribution statement

All authors listed have significantly contributed to the development and the writing of this article.

### Funding statement

This research did not receive any specific grant from funding agencies in the public, commercial, or not-for profit sectors.

### Data availability statement

Data will be made available on request.

### Declaration of interest’s statement

The authors declare no conflict of interest.

### Additional information

No additional information is available for this paper.
